# A neutral view of the evolving genomic architecture of speciation

**DOI:** 10.1002/ece3.3190

**Published:** 2017-07-06

**Authors:** Laura Southcott, Marcus R. Kronforst

**Affiliations:** ^1^ Committee on Evolutionary Biology University of Chicago Chicago IL USA; ^2^ Department of Ecology and Evolution University of Chicago Chicago IL USA

**Keywords:** *F*_ST_ outlier loci, gene flow, genome scans, phylogenomics

## Abstract

Analyses of genomewide polymorphism data have begun to shed light on speciation and adaptation. Genome scans to identify regions of the genome that are unusually different between populations or species, possibly due to divergent natural or sexual selection, are widespread in speciation genomics. Theoretical and empirical work suggests that such outlier regions may grow faster than linearly during speciation with gene flow due to a rapid transition between low and high reproductive isolation. We investigate whether this pattern could be attributed to neutral processes by simulating genomes under neutral evolution with varying amounts and timing of gene flow. Under both neutral evolution and divergent selection, simulations with little or no gene flow, or with a long allopatric period after its cessation, resulted in faster than linear growth of the proportion of the genome lying in outlier regions. Without selection, higher recent gene flow erased differentiation; with divergent selection, these same scenarios produced nonlinear growth to a plateau. Our results suggest that, given a history of gene flow, the growth of the divergent genome is informative about selection during divergence, but that in many scenarios, this pattern does not easily distinguish neutral and non‐neutral processes during speciation with gene flow.

## 1 INTRODUCTION

Speciation is responsible for the diversity of life on earth, and understanding its mechanisms is of great interest to evolutionary biologists. A genetic approach to this field seeks to identify loci driving speciation and characterize the changing patterns of divergence across the genome during speciation. Technological progress in genetics and genomics is rapidly advancing this research program (Seehausen et al., 2014; Twyford & Ennos, 2012). Recently, the availability of whole genome sequences and reduced representation genomic data in an increasing variety of species has begun to make tests of predictions concerning genomewide patterns of divergence possible (Gagnaire, Pavey, Normandeau, & Bernatchez, 2013; Renaut et al., 2013; Roesti, Hendry, Salzburger, & Berner, 2012; Soria‐Carrasco et al., 2014).

An increasingly accepted view is that genomic divergence between closely related species is often heterogeneous (Feder, Egan, & Nosil, 2012; Nosil, Funk, & Ortiz‐Barrientos, 2009; Rieseberg & Burke, 2001; Wu, 2001). Certain genomic regions, namely those tightly linked to loci causing reproductive isolation between occasionally hybridizing taxa, may be especially resistant to gene flow, while unlinked neutral differences easily introgress between species. When selection is strong and populations are small, the region linked to the selected locus can increase in size via divergence hitchhiking (Feder & Nosil, 2010; Via & West, 2008). At the same time, strong reproductive isolation promotes divergence across the whole genome (Nosil & Feder, 2012); during ecological speciation, the loci underlying reproductive isolation experience divergent natural selection (Schluter, 2009).

Methods to identify regions of the genome that contain targets of divergent selection between species make use of the population genetic prediction that loci subject to positive selection will exhibit low within‐population variance but high between‐population variance when compared to loci that have not undergone the same selection regime. Thus, genome scans in which a metric of population differentiation such as *F*_ST_ is calculated for consecutive sequence windows or for markers dispersed across the genome can be used to identify areas with unusually high differentiation (speciation islands), areas which should be enriched for genes causing reproductive isolation between taxa (Turner, Hahn, & Nuzhdin, 2005). Such methods have limits and may be unsuitable when used alone to infer differential gene flow among genomic regions (Burri et al., 2015; Cruickshank & Hahn, 2014; Wolf & Ellegren, 2016). Indeed, a suite of processes other than divergent selection on two alternative alleles can create peaks in the *F*_ST_ landscape (Bierne, 2010; Exocoffier & Ray, 2008; Roesti, Gavrilets, Hendry, Salzburger, & Berner, 2014), and the power to detect outliers may vary across the genome (Cruickshank & Hahn, 2014). However, outlier scans provide useful guides for identifying loci involved in species differences and thus have become a standard tool in speciation genomics (Beaumont, 2005; Strasburg et al., 2012; Wolf & Ellegren, 2016).

While outlier analyses were initially used to compare two populations or species, a new goal is to compare outlier scans among multiple pairs of species to investigate parallel adaptation/speciation and divergence at different stages and in different demographic situations. For example, four independently evolved pairs of host‐plant races of the stick insect *Timema cristinae* exhibited parallel divergence in some regions of the genome, but other highly differentiated regions were unique to each population pair (Soria‐Carrasco et al., 2014). All *T. cristinae* host‐race pairs in Soria‐Carrasco et al. (2014) were young and had high gene flow, but areas of high divergence, as determined by a hidden Markov model, comprised 8%–30% of the genome, with the largest percentage found in the one pair that was geographically separated rather than adjacent.

Other studies have begun to compare genome‐wide differentiation patterns across stages of divergence. In lake and stream threespine stickleback (*Gasterosteus aculeatus*), the median genomewide *F*_ST_ was higher in pairs with greater morphological differences (Roesti et al., 2012). Independently evolved pairs of dwarf and normal lake whitefish (*Coregonus clupeaformis*) also had higher overall *F*_ST_ when they were more phenotypically divergent (Gagnaire et al., 2013), and increasingly genetically differentiated population pairs had more large divergent regions (Renaut et al., 2012). Highly divergent regions comprised a smaller portion of the genome in comparisons of sunflower ecotypes than in comparisons between species (Andrew & Rieseberg, 2013). Comparisons among several sunflower species, however, found that the size and number of divergent regions differed little among pairs in different geographic (and gene flow) contexts (Renaut et al., 2013). On the other hand, when three phylogenetically independent species pairs were compared, mean SNP *F*_ST_ was larger in older species pairs (Renaut, Owens, & Rieseberg, 2014).

Currently, there is relatively little theoretical work that explores expected genomewide patterns during divergence with gene flow. Flaxman, Wacholder, Feder, and Nosil (2014) found that, for certain combinations of migration rate and strength of selection, the effective migration rate (a measure of reproductive isolation) decreases gradually at first, then sharply. This rate change coincided with an abrupt change in the rate of increase of divergently selected loci. Thus, during speciation with gene flow the genome may transition from a porous phase characterized by free gene flow of alleles that are not under divergent selection to a phase dominated by reproductive isolation across the whole genome and widespread linkage disequilibrium. This process has been termed “genomewide congealing” (Feder et al., 2014; Flaxman et al., 2014). The genomewide congealing hypothesis joins other theoretical predictions of nonlinear dynamics during speciation with gene flow. Adaptive dynamics suggests that speciation can occur abruptly due to disruptive selection at certain points in bivariate trait space (Geritz et al., 1998; Ito & Dieckmann, 2012). Coupling of incompatibility loci, under certain conditions, feeds back to cause the evolution of further coupling, leading to nonlinear transitions across hybrid zones and over evolutionary time (Barton, 1983; Barton & Bengtsson, 1986; Barton & De Cara, 2009; Bierne, Welch, Loire, Bonhomme, & David, 2011). However, nonlinearity even without selection and gene flow may occur. The snowball theory, for instance, predicts a faster than linear increase in the number of genetic incompatibilities as a result of epistatic interactions among linearly increasing substitutions (Orr, 1995). Outlier scans examine the distribution of these linearly increasing substitutions without considering their effects (including epistatic effects). Under genomewide congealing, nonlinear increases in outliers might be expected. Thus, the behavior of allopatrically diverging populations in the absence of selection should be considered for comparison with the predictions of genomewide congealing models.

To quantify how genomewide divergence patterns in nature change with time since speciation, Kronforst et al. (2013) examined divergence between three species of *Heliconius* butterflies from Costa Rica. They found that the proportion of the genome that lay in highly divergent regions increased faster than linearly with increasing time since divergence between each pair of species. They attributed this result to gradually attenuating gene flow during speciation, with a tipping point hybridization rate above which divergence is inhibited and below which it accelerates—a suggestion similar to a genomic “congealing” process. However, Flaxman et al. (2014) predicted speciation in *Heliconius* to proceed without such a nonlinear transition, because it often involves few genes of large effect (Kronforst & Papa, 2015; Nadeau et al., 2012).

To explore whether this faster than linear increase could be produced by processes other than selection interacting with gene flow, we simulated neutral evolution in allopatry and compared it to various scenarios of speciation with gene flow and selection and to Kronforst et al.'s (2013) results for *Heliconius*. Our results suggest next steps in examining genomewide patterns of divergence and highlight the need for null model comparisons in the emerging field of speciation genomics.

## 2 METHODS

### 2.1 Simulations

**Figure 1:**
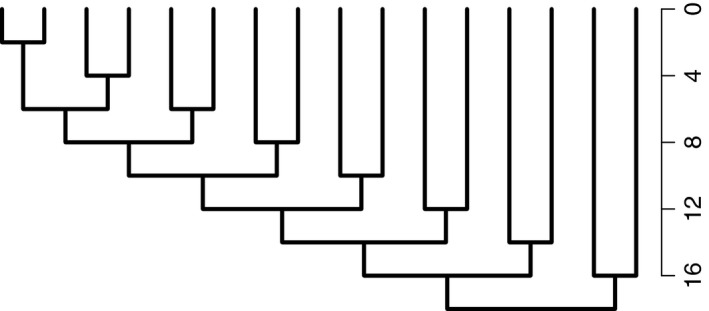
Relationships among species simulated in this study. *F*_ST_ was only calculated between sister species to avoid phylogenetic nonindependence. Scale is in units of *N* generations, where *N* is the effective population size of 10^6^

We generated gene trees using the neutral coalescent modeling software ms (Hudson, 2002), then evolved sequences along these trees with Seq‐Gen (Rambaut & Grassly, 1997) under a Jukes–Cantor model. We simulated three scenarios: no gene flow, gene flow between sister species for the first 2 *N* generations following divergence (“early gene flow”), and gene flow for the most recent 2 *N* generations (“recent gene flow”), where *N* is the effective population size. These scenarios correspond to allopatric speciation, speciation with gene flow followed by complete reproductive isolation, and secondary contact following allopatric divergence respectively. We simulated each scenario 30 times, and each time simulated ten sequences from each of 16 species comprising eight sister pairs of varying ages (from 2 *N* to 16 *N* generations; Figure 1). Each species had the same constant effective population size (*N*); we simulated *N* = 10^6^, 10^5^, and 10^4^. Each sequence was 100 kbp long with a recombination rate of 10^−8^ per site per generation and a mutation rate of 5 × 10^−9^ per site per generation. Because of the short genome length, we did not attempt to vary recombination or mutation rate within the genome, nor could we investigate long‐range linkage disequilibrium. For *N* = 10^6^, we simulated both unidirectional and bidirectional gene flow with migration parameters of 4 Nm = 10, 1, 0.1, 0.01, and 0.001. For *N* = 10^5^, we simulated unidirectional migration of 4 Nm = 0.0001, 0.01, and 1; and for *N* = 10^4^, 4 Nm = 0.00001, 0.001, and 0.1. These correspond to the same migration rates (m) as simulations of *N* = 10^6^ and 4 Nm = 0.001, 0.1, and 10.

For a subset of these demographic scenarios (*N* = 10^6^ and no gene flow, unidirectional early gene flow with 4 Nm = 10, and unidirectional recent gene flow with 4 Nm = 0.001, 0.01, 0.1, 1.0, and 10.0), we also investigated gene trees for regions adjacent to a selected locus using msms (Ewing & Hermisson, 2010), a coalescent simulator based on ms that incorporates forward‐in‐time simulations of selection on single loci. We modeled gene trees for 100 kb adjacent to a selected locus with two alleles under divergent selection between species (*s* = 0.01 or 0 for homozygotes and *s* = 0.005 for heterozygotes). As for the neutral simulations, we generated sequences with Seq‐Gen. The full ms/msms and Seq‐Gen input parameters are presented in the Supporting Information.

### 2.2 Analysis

We used the package PopGenome (Pfeifer, Wittelsbürger, Ramos‐Onsins, & Lercher, 2014) in R (R Core Team 2013) version 2.15.0 to calculate *F*_ST_ using polymorphic loci (Hudson, Slatkin, & Maddison, 1992) between sister species for each nonoverlapping 500 bp window in the simulated genomes. For each run of the simulation (160 sequences from eight species pairs), all *F*_ST_ values were pooled to set a common threshold for identifying outliers across species. We considered the 80th, 95th, and 99th percentiles as thresholds above which an *F*_ST_ value was considered an outlier. We also applied a relaxed threshold method in which, if all intervening windows between two consecutive 95% outlier windows had *F*_ST_ larger than the 75th percentile, these windows were also designated outliers (Kronforst et al., 2013). We also calculated average between‐population nucleotide divergence (*d*_*xy*_, Nei, 1987) and number of fixed differences for each species pair.

For each simulation run, we performed linear and exponential regressions, both forced through the origin, of divergent window number versus time since divergence of the species pair. We compared AIC values for the two regressions. As all scenarios except recent gene flow with a migration rate of 1 or 10 clearly followed an exponential rather than linear curve, we compared the coefficients of the exponential regression (*b* in *y *= *a*(1 − *e* ^*bx*^)) among gene flow scenarios with an ANOVA.

## 3 RESULTS

### 3.1 No selection

**Figure 2:**
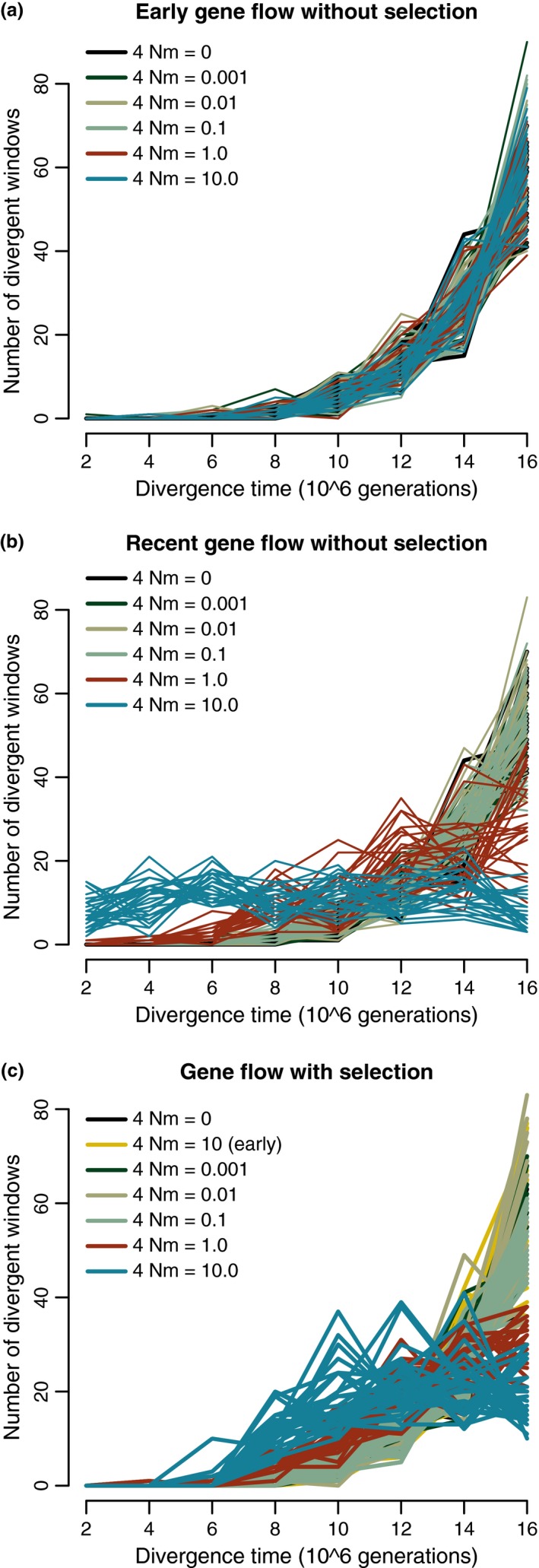
(a) Divergent genome size (number of outlier windows) increases faster than linearly with divergence time when no selection and no gene flow or various levels of unidirectional early gene flow occurs. (b) The highest levels of unidirectional recent gene flow homogenize the genome and prevent this increase. (c) The same pattern occurs in simulations with divergent selection and no gene flow, high gene flow, or recent gene flow up to 4 Nm = 0.1. Higher gene flow (4 Nm = 1.0 or 10.0) and divergent selection result in a nonlinear increase to a plateau. Each line represents a single simulation of eight between‐species comparisons

Results for unidirectional gene flow and *N* = 10^6^ are presented here; scenarios with bidirectional gene flow did not differ substantially for the input parameters we tested and are presented in the Supporting Information (Fig. S1). The number of divergent windows increased faster than linearly when no gene flow or early gene flow up to 4 Nm = 10 occurred (Figure 2), and an exponential function fit the data better than a linear regression in each simulation (ΔAIC = 7.36 − 59.21). The faster than linear increase was found regardless of the *F*_ST_ outlier threshold (Fig. S2). The rest of the results presented use a 95% + 75% *F*_ST_ threshold, as described in Section 2.

In the recent gene flow scenario, exponential curves fit the data better than linear functions for migration parameters of 0.001, 0.01, and 0.1 (ΔAIC = 3.35–49.61). For 4 Nm = 10, nonlinear regressions did not converge after 1,000 iterations for any of the simulated datasets. For 4 Nm = 1, nonlinear regression converged in only 10 of 29 simulated datasets.

**Figure 3:**
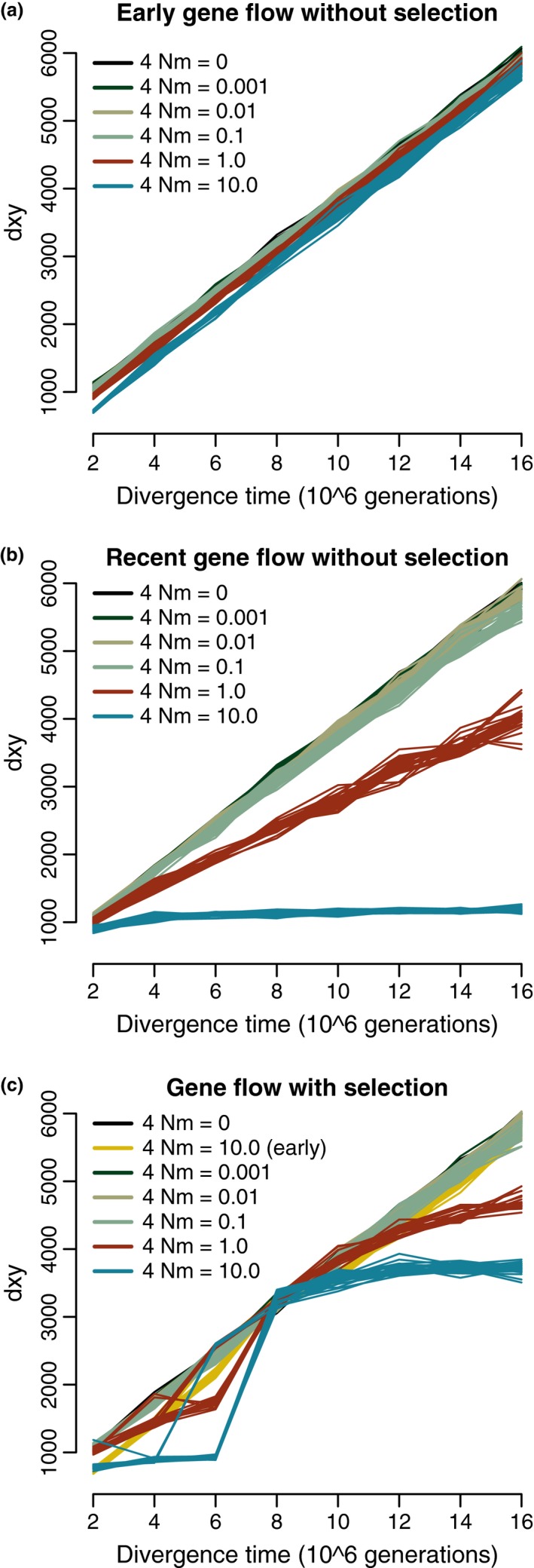
(a) For simulations without selection, average pairwise substitutions (*d*_*xy*_) increase linearly with divergence time with early gene flow. (b) A less rapid increase occurs with higher recent gene flow. (c) With selection, *d*_*xy*_ increases linearly when gene flow is absent, early, or minimal and recent, but increases nonlinearly with higher recent gene flow

In all scenarios, *d*_*xy*_ increased linearly, more rapidly when there was less migration (Figure 3). The number of fixed differences between species pairs increased linearly with no or early gene flow, but began to plateau when higher recent gene flow was considered (Fig. S3). Both global and mean per window *F*_ST_ increased at a decelerating rate in the no gene flow scenario, as expected from coalescent theory (Supplemental Results), and stayed approximately constant over increasing divergence time in the 4 Nm = 10 recent gene flow scenario (Fig. S4–S8).

**Figure 4:**
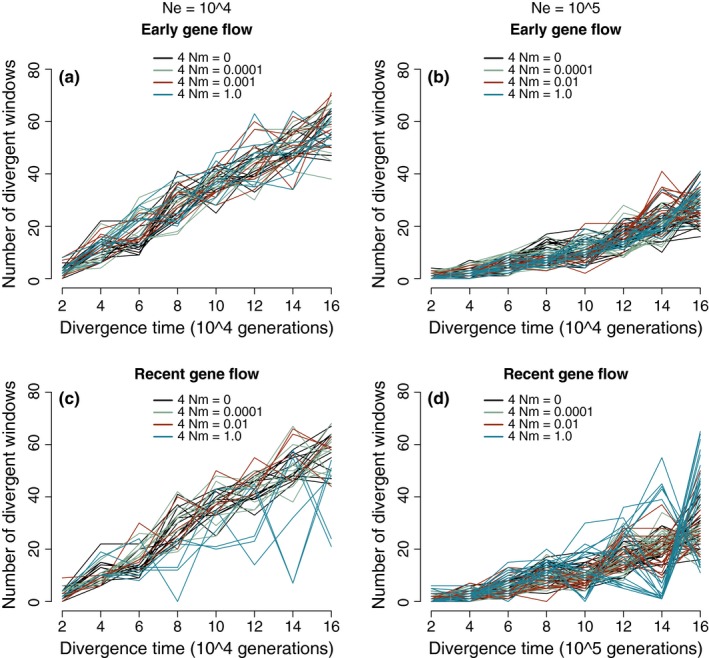
Patterns of change in divergent genome size over time with smaller effective population sizes. (a) *N* = 10^4^, early migration (for the first 2 *N* generations after divergence). (b) *N* = 10^5^, early migration. (c) *N* = 10^4^, recent migration (for the most recent 2 *N* generations). (d) *N* = 10^5^, recent migration. Note that the *x*‐axis is scaled to population size, and thus differ by an order of magnitude between a/c and b/d

Smaller population sizes and correspondingly shorter divergence times differed strikingly from simulations with *N* = 10^6^. When *N* = 10^4^, the divergent genome grew linearly (Figure 4), perhaps because divergence was more rapid than in larger populations and *F*_ST_ approached 1 closely by the oldest time since divergence. The highest recent gene flow level (4 Nm = 0.1) did not completely homogenize the diverging populations. At *N* = 10^5^, divergent genome size increased nonlinearly, but less steeply than in simulations of *N* = 10^6^ (Figure 4). At these lower population sizes, there was again little difference between no migration, early migration, and limited recent migration scenarios.

### 3.2 Divergent selection

Our simulations with a single divergently selected locus produced a nonlinear increase when there was no gene flow or early gene flow (4 Nm = 10). For both scenarios, exponential functions fit the data better than linear regression (ΔAIC = 5.34–50.49). This was also the case for scenarios with selection and recent gene flow up to 4 Nm = 0.1 (ΔAIC = 2.76–49.55; Figure 2). However, selection with recent gene flow of 4 Nm = 1 or 10 resulted in a gradual increase in divergent genome size that appeared to plateau, as in a logistic function. For 4 Nm = 10, nonlinear regressions did not converge in any simulated datasets, and for 4 Nm = 1, they converged in only 12 of 21 datasets.

The number of fixed differences and *d*_*xy*_ increased linearly for all scenarios except recent gene flow with 4 Nm = 1 or 10. In these scenarios, both statistics underwent a rapid increase between 4 × 10^6^ and 8 × 10^6^ generations followed by a more gradual increase or, for 4 Nm = 10, an apparent plateau (Figure 3 and Fig. S3).

**Figure 5:**
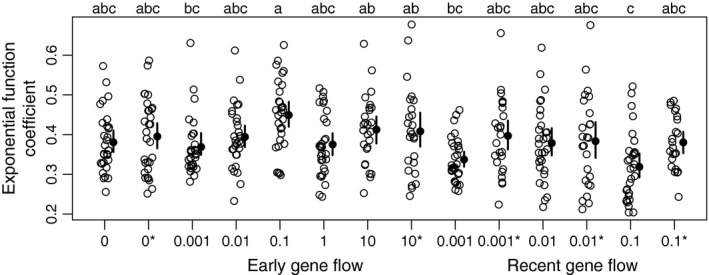
The rate of increase in divergent genome size (the coefficient in the exponential function) depends on the extent of recent gene flow. Migration parameter (4 Nm) is on the *x*‐axis; values with asterisks represent simulations with selection. Open circles are individual data points; filled circles are means with bias‐corrected bootstrap 95% confidence intervals. Scenarios that do not share the same letter (top) are significantly different based on Tukey's honestly significant difference test

When exponential curves were fit to each scenario (both with and without selection, and excluding those with recent gene flow of 4 Nm = 10 or 1 due to nonconvergence), the rate of increase of divergent windows versus time (the coefficient of divergence time in the exponential equation) differed among scenarios (ANOVA, *F* = 3.89, *p* = 6.1 × 10^−6^). However, based on Tukey's HSD test, no scenario differed from the no gene flow, no selection simulations (mean = 0.38, *SD* = 0.08); only early gene flow of 4 Nm = 0.1 without selection (mean = 0.45, *SD* = 0.09) and recent gene flow of 4 Nm = 0.1 without selection (mean = 0.32, *SD* = 0.09) significantly differed from each other (Figure 5).

## 4 DISCUSSION

Our results show that, in many demographic scenarios, an exponential increase in divergent genome size provides no evidence for or against gene flow and selection during speciation. When gene flow is extensive and recent, however, divergent selection produces a distinct pattern in both the growth of outlier regions and the increase in *d*_*xy*_, and thus in situations with known gene flow these statistics could provide insight into the action of selection. Likewise, our simulations of smaller populations with shorter divergence times suggest that some neutral scenarios produce linear increases, and thus, a nonlinear increase with these demographics might be a useful signature of speciation with gene flow. Below we discuss the implications of our findings for comparative studies of genomewide divergence and the strengths and weaknesses of this method of outlier analysis.

The *Heliconius* species that inspired this study showed a nonlinear increase in the divergent genome and in the number of fixed differences between species but linear growth of *d*_*xy*_ (Kronforst et al., 2013). These results were interpreted as evidence for an interplay between selection and gene flow during speciation, but we did not find this specific combination of patterns in the scenarios we simulated. Our results show that exponential growth of the divergent genome occurs even without selection and gene flow while *d*_*xy*_ and the number of fixed differences mirror one another and only show nonlinear behavior with selection and gene flow. The disconnect may stem from the fact that *Heliconius* does not closely match the simulation parameters—population size estimates range from 250,000 to 1.8 million across the three *Heliconius* species and divergence times go back an estimated 6 million generations (Kronforst et al., 2013), which is an early time point for the *N* = 10^6^ simulations but extremely old (off the charts) for the *N* = 10^5^ simulations. However, ample evidence exists that *Heliconius* species are under divergent natural selection, especially on wing color pattern, and that hybridization occurs among taxa (Bull et al., 2006; Heliconius Genome Consortium 2012; Kronforst, 2008; Kronforst, Young, Blume, & Gilbert, 2006; Mallet, Beltrán, Neukirchen, & Linares, 2007; Martin et al., 2013; Nadeau et al., 2013; Wu, Joron, & Jiggins, 2010; Zhang, Dasmahapatra, Mallet, Moreira, & Kronforst, 2016). Furthermore, Kronforst et al. (2013) estimated migration parameters (4 Nm) ranging from approximately 1 to well over 10. We might conclude in this case that while the exponential growth of the divergent genome is not in itself informative, the behavior of the number of fixed differences in *Heliconius* does support the involvement of selection and gene flow during speciation.

The nonlinear increase in divergent genome size in our simulations is reminiscent of predictions that ecological speciation with gene flow proceeds rapidly once a threshold number of divergently selected mutations has accumulated (Feder et al., 2014; Flaxman et al., 2014). Flaxman et al.'s (2014) simulations of this process found that it occurred under a wide range of mean selection coefficients and migration rates. However, they also cautioned that scenarios other than speciation with gene flow could produce such a pattern (Feder et al., 2014; Flaxman et al., 2014). We found some evidence for a “congealing”‐like process in simulations with divergent selection and high recent gene flow, in that *d*_*xy*_ exhibited a rapid increase between 4 and 8 million generations. Further examination will be needed to determine under what conditions this pattern occurs and whether it reliably indicates divergence with selection and gene flow. However, nonlinear growth of *F*_ST_ outlier regions in many scenarios that lacked selection indicates that this pattern cannot be attributed to “congealing”‐like processes. While Flaxman et al.'s (2014) results and ours are not directly comparable—the former used an individual‐based forward simulation of only loci that differed between species while we employed a coalescent simulation of a fixed genome size—both studies suggest that characterization of “congealing”‐like processes in allopatrically diverging populations, both with and without selection, is the next step in the maturation of speciation genomics as a field. Theoretical work like this is important for understanding emerging results from empirical studies of genomic divergence across multiple speciation events.

Outlier loci are detected in many ways (Andrew & Rieseberg, 2013; Gagnaire et al., 2013; Renaut et al., 2013; Soria‐Carrasco et al., 2014; Turner et al., 2005). Our method—using an arbitrary high percentile of the *F*_ST_ distribution of all windows—is a particularly rough heuristic, in that it finds areas of the genome that should be enriched for loci under divergent selection, but it will do so even if no selection has occurred, as in these simulations. As applying this method to data from wild populations will detect both selected and neutral loci, it is necessary to understand how the method treats purely neutral loci. Additionally, our simulations that included divergent selection only modeled selection on a single locus and, due to computational limits, only examined a small (100 kb) neutral region linked to it. More complex genetic architectures of selected loci could produce radically different patterns of genomic divergence, but would require a different modeling approach to simulate.

The choice of *F*_ST_ outlier threshold is arbitrary, and we found qualitatively similar results with different thresholds (Fig. S2). What is key for our purposes is that we set the same absolute threshold for all comparisons between species (Kronforst et al., 2013). Setting a different threshold for each species pair would remove the effects of divergence time on *F*_ST_ and thus make it impossible to study the relationship between divergence time and the proportion of loci in highly diverged regions, the sort of study required to look for genomewide congealing (Feder et al., 2014). Other recent studies have compared the position of outlier regions among parallel species pairs in different geographic contexts and gene flow scenarios, largely without considering divergence time (Gagnaire et al., 2013; Renaut et al., 2013, 2014; Roesti et al., 2012; Soria‐Carrasco et al., 2014). In such comparisons, setting separate thresholds for each species/population pair is appropriate. However, to look for changes in the proportion of the genome that is highly divergent over time, it is necessary to set a single threshold.

Our findings of an exponential increase in low gene flow scenarios may follow from applying a single threshold. Statistically, applying a single threshold to the extreme tail of a pool of overlapping normal distributions results in an exponential increase, because this combined tail is dominated by values from the distributions with the furthest offset means (in our case, the older species pairs; Fig. S9). This phenomenon may thus underlie our findings for low gene flow scenarios (Figs S5, S7). However, high gene flow alters the variance and/or skew of the *F*_ST_ distributions and reduces the offset among their means (Figs S6, S8), producing different patterns of outlier region growth.

In our simulations, each species pair was subject to the same demography, gene flow, and selection scenario, differing only by divergence time. Having different pairs of species experience different amounts and timing of gene flow, allowing changes in population sizes, and incorporating other more complex demographic histories and variation in recombination rates (including variation within a genome) would further change these distributions, and make the relationship between gene flow and the size of the divergent regions less clear. Different durations of gene flow are likely to have a large impact on the growth of outlier regions because of gene flow's homogenizing effect in the absence of selection. While methods exist to determine the demographic history of related populations (Becquet & Przeworski, 2009; Hey & Nielsen, 2004; Nielsen & Wakeley, 2001), and one could then simulate neutral evolution based on that demography to which real data could be compared, such simulations are computationally intractable for large linkage groups. Likewise, taxa with different tree topologies would require corrections for phylogenetic nonindependence before these comparisons could be made (Felsenstein, 1985). Nonetheless, examining the raw distributions of *F*_ST_ per window could allow greater insight in more complicated demographic scenarios.

Our findings suggest some first steps for examining divergence at a genomewide scale in comparative studies. The growth of outlier regions provides information about whether selection acted during speciation only under some circumstances, specifically when extensive gene flow is known to have occurred. This pattern combined with changes in *d*_*xy*_ between species pairs warrants further study as an indicator of speciation with gene flow. Finally, our findings reinforce the fact that not all patterns found in species that are known to experience divergence with gene flow and/or selection are characteristic of divergence with gene flow or selection. To make such claims, null models are necessary for comparison, but they require estimates of demography (which may be unreliable) and massive computational power. Refinement of these techniques offers great promise to look at genomewide changes during speciation.

## CONFLICT OF INTEREST

None declared.
