# Supplementary material for: A neutral view of the evolving genomic architecture of speciation
Source: Ecol Evol. 2017 Jul 6;7(16):6358–66. doi: 10.1002/ece3.3190 (PMC5574762; doi:10.1002/ece3.3190)
Supplement: Supplementary file 1 [file ECE3-7-6358-s001.pdf]

Supplemental material for: A neutral view of the evolving genomic architecture of speciation

January 9, 2017

Simulation Parameters

The following are the input codes for ms (or msms) and Seq-Gen for each simulation scenario with an effective population size of 10^6. The full code for sequence generation and analysis is available from the Dryad data repository.

No selection

No gene flow

```
./ms 160 1 -T -r 4000 100000 -I 16 10 10 10 10 10 10 10 10 10 10
    10 10 10 10 10 10 -ej .5 1 2 -en .5 2 .5 -ej 1 3 4 -en 1 4 .5 -
    ej 1.5 2 4 -en 1.5 4 .5 -ej 1.5 5 6 -en 1.5 6 .5 -ej 2 4 6 -en
    2 6 .5 -ej 2 7 8 -en 2 8 .5 -ej 2.5 6 8 -en 2.5 8 .5 -ej 2.5 9
    10 -en 2.5 10 .5 -ej 3 8 10 -en 3 10 .5 -ej 3 11 12 -en 3 12 .5
     -ej 3.5 10 12 -en 3.5 12 .5 -ej 3.5 13 14 -en 3.5 14 .5 -ej 4
    12 14 -en 4 14 .5 -ej 4 15 16 -en 4 16 .5 -ej 4.5 14 16 -en 4.5
     16 .5
```

```
./Seq-Gen.v1.3.3/source/seq-gen -mHKY -l 100000 -s .02 -p 483605
```

Early gene flow

```
./ms 160 1 -T -r 4000 100000 -I 16 10 10 10 10 10 10 10 10 10 10
    10 10 10 10 10 10 -m 2 1 10.0 -ej .5 1 2 -en .5 2 .5 -em .5 4 3
    10.0 -ej 1 3 4 -en 1 4 .5 -em 1 6 5 10.0 -ej 1.5 2 4 -en 1.5 4
    .5 -ej 1.5 5 6 -en 1.5 6 .5 -em 1.5 8 7 10.0 -ej 2 4 6 -en 2 6
    .5 -ej 2 7 8 -en 2 8 .5 -em 2 10 9 10.0 -ej 2.5 6 8 -en 2.5 8
    .5 -ej 2.5 9 10 -en 2.5 10 .5 -em 2.5 12 11 10.0 -ej 3 8 10 -en
    3 10 .5 -ej 3 11 12 -en 3 12 .5 -em 3 14 13 10.0 -ej 3.5 10 12
    -en 3.5 12 .5 -ej 3.5 13 14 -en 3.5 14 .5 -em 3.5 16 15 10.0 -
    ej 4 12 14 -en 4 14 .5 -ej 4 15 16 -en 4 16 .5 -ej 4.5 14 16 -
    en 4.5 16 .5
```

```
./Seq-Gen.v1.3.3/source/seq-gen -mHKY -l 100000 -s .02 -p 483605
```

Recent gene flow

```
./ms 160 1 -T -r 4000 100000 -I 16 10 10 10 10 10 10 10 10 10 10
    10 10 10 10 10 10 -m 2 1 1 -m 4 3 1 -m 6 5 1 -m 8 7 1 -m 10 9 1
    -m 12 11 1 -m 14 13 1 -m 16 15 1 -eM .5 0 -ej .5 1 2 -en .5 2
    .5 -ej 1 3 4 -en 1 4 .5 -ej 1.5 2 4 -en 1.5 4 .5 -ej 1.5 5 6 -
    en 1.5 6 .5 -ej 2 4 6 -en 2 6 .5 -ej 2 7 8 -en 2 8 .5 -ej 2.5 6
    8 -en 2.5 8 .5 -ej 2.5 9 10 -en 2.5 10 .5 -ej 3 8 10 -en 3 10
    .5 -ej 3 11 12 -en 3 12 .5 -ej 3.5 10 12 -en 3.5 12 .5 -ej 3.5
    13 14 -en 3.5 14 .5 -ej 4 12 14 -en 4 14 .5 -ej 4 15 16 -en 4
    16 .5 -ej 4.5 14 16 -en 4.5 16 .5
```

```
./Seq-Gen.v1.3.3/source/seq-gen -mHKY -l 100000 -s .02 -p 483605
```

The migration parameter (4Nm, the third value after the -m switches) was set to 0.001, 0.01, 0.1, 1, or 10 for simulations with a population size of 10^6. For population sizes of 10^5, migration parameters were 0.0001, 0.01, and 1; for N = 10^4, they were 0.00001, 0.001, and 0.1. These migration parameters have the same migration proportions as 4Nm = 0.001, 0.1, and 10 for N = 10^6.

Divergent selection

No gene flow

```
./msms 160 1 -T -r 4000 100000 -N 1000000 -I 16 10 10 10 10 10 10
    10 10 10 10 10 10 10 10 10 10 -ej .5 1 2 -en .5 2 .5 -ej 1 3 4
    -en 1 4 .5 -ej 1.5 2 4 -en 1.5 4 .5 -ej 1.5 5 6 -en 1.5 6 .5 -
    ej 2 4 6 -en 2 6 .5 -ej 2 7 8 -en 2 8 .5 -ej 2.5 6 8 -en 2.5 8
    .5 -ej 2.5 9 10 -en 2.5 10 .5 -ej 3 8 10 -en 3 10 .5 -ej 3 11
    12 -en 3 12 .5 -ej 3.5 10 12 -en 3.5 12 .5 -ej 3.5 13 14 -en
    3.5 14 .5 -ej 4 12 14 -en 4 14 .5 -ej 4 15 16 -en 4 16 .5 -ej
    4.5 14 16 -en 4.5 16 .5 -SI 2 16 .5 .5 .5 .5 .5 .5 .5 .5 .5 .5
    .5 .5 .5 .5 .5 .5 -Sc 0 16 0 100000 200000 -Sc 0 15 200000
    100000 0 -Sc 0 14 0 100000 200000 -Sc 0 13 200000 100000 0 -Sc
    0 12 0 100000 200000 -Sc 0 11 200000 100000 0 -Sc 0 10 0 100000
     200000 -Sc 0 9 200000 100000 0 -Sc 0 8 0 100000 200000 -Sc 0 7
     200000 100000 0 -Sc 0 6 0 100000 200000 -Sc 0 5 200000 100000
    0 -Sc 0 4 0 100000 200000 -Sc 0 3 200000 100000 0 -Sc 0 2 0
    100000 200000 -Sc 0 1 200000 100000 0 -Sc .5 2 0 0 0 -Sc 1 4 0
```

```
0 0 −Sc 1.5 6 0 0 0 −Sc 2 8 0 0 0 −Sc 2.5 10 0 0 0 −Sc 3 12 0 0
  0 −Sc 3.5 14 0 0 0 −Sc 4 16 0 0 0

./Seq−Gen.v1.3.3/source/seq−gen −mHKY −l 100000 −s .02 −p 483605
```

Early gene flow

```
./msms 160 1 −T −r 4000 100000 −N 1000000 −I 16 10 10 10 10 10 10
  10 10 10 10 10 10 10 10 10 10 −m 2 1 10.0 −ej .5 1 2 −en .5 2
  .5 −em .5 4 3 10.0 −ej 1 3 4 −en 1 4 .5 −em 1 6 5 10.0 −ej 1.5
  2 4 −en 1.5 4 .5 −ej 1.5 5 6 −en 1.5 6 .5 −em 1.5 8 7 10.0 −ej
  2 4 6 −en 2 6 .5 −ej 2 7 8 −en 2 8 .5 −em 2 10 9 10.0 −ej 2.5 6
  8 −en 2.5 8 .5 −ej 2.5 9 10 −en 2.5 10 .5 −em 2.5 12 11 10.0 −
  ej 3 8 10 −en 3 10 .5 −ej 3 11 12 −en 3 12 .5 −em 3 14 13 10.0
  −ej 3.5 10 12 −en 3.5 12 .5 −ej 3.5 13 14 −en 3.5 14 .5 −em 3.5
  16 15 10.0 −ej 4 12 14 −en 4 14 .5 −ej 4 15 16 −en 4 16 .5 −ej
  4.5 14 16 −en 4.5 16 .5 −SI 2 16 .5 .5 .5 .5 .5 .5 .5 .5 .5 .5
  .5 .5 .5 .5 .5 .5 −Sc 0 16 0 100000 200000 −Sc 0 15 200000
  100000 0 −Sc 0 14 0 100000 200000 −Sc 0 13 200000 100000 0 −Sc
  0 12 0 100000 200000 −Sc 0 11 200000 100000 0 −Sc 0 10 0 100000
  200000 −Sc 0 9 200000 100000 0 −Sc 0 8 0 100000 200000 −Sc 0 7
  200000 100000 0 −Sc 0 6 0 100000 200000 −Sc 0 5 200000 100000
  0 −Sc 0 4 0 100000 200000 −Sc 0 3 200000 100000 0 −Sc 0 2 0
  100000 200000 −Sc 0 1 200000 100000 0 −Sc .5 2 0 0 0 −Sc 1 4 0
  0 0 −Sc 1.5 6 0 0 0 −Sc 2 8 0 0 0 −Sc 2.5 10 0 0 0 −Sc 3 12 0 0
  0 −Sc 3.5 14 0 0 0 −Sc 4 16 0 0 0
```

```
./Seq-Gen.v1.3.3/source/seq-gen -mHKY -l 100000 -s .02 -p 483605
```

Recent gene flow

```
./msms 160 1 -T -r 4000 100000 -N 1000000 -I 16 10 10 10 10 10 10
  10 10 10 10 10 10 10 10 10 10 -m 2 1 10.0 -m 4 3 10.0 -m 6 5
  10.0 -m 8 7 10.0 -m 10 9 10.0 -m 12 11 10.0 -m 14 13 10.0 -m 16
  15 10.0 -eM .5 0 -ej .5 1 2 -en .5 2 .5 -ej 1 3 4 -en 1 4 .5 -
  ej 1.5 2 4 -en 1.5 4 .5 -ej 1.5 5 6 -en 1.5 6 .5 -ej 2 4 6 -en
  2 6 .5 -ej 2 7 8 -en 2 8 .5 -ej 2.5 6 8 -en 2.5 8 .5 -ej 2.5 9
  10 -en 2.5 10 .5 -ej 3 8 10 -en 3 10 .5 -ej 3 11 12 -en 3 12 .5
  -ej 3.5 10 12 -en 3.5 12 .5 -ej 3.5 13 14 -en 3.5 14 .5 -ej 4
  12 14 -en 4 14 .5 -ej 4 15 16 -en 4 16 .5 -ej 4.5 14 16 -en 4.5
  16 .5 -SI 2 16 .5 .5 .5 .5 .5 .5 .5 .5 .5 .5 .5 .5 .5 .5 .5 .5
  -Sc 0 16 0 100000 200000 -Sc 0 15 200000 100000 0 -Sc 0 14 0
  100000 200000 -Sc 0 13 200000 100000 0 -Sc 0 12 0 100000 200000
  -Sc 0 11 200000 100000 0 -Sc 0 10 0 100000 200000 -Sc 0 9
  200000 100000 0 -Sc 0 8 0 100000 200000 -Sc 0 7 200000 100000 0
  -Sc 0 6 0 100000 200000 -Sc 0 5 200000 100000 0 -Sc 0 4 0
  100000 200000 -Sc 0 3 200000 100000 0 -Sc 0 2 0 100000 200000 -
  Sc 0 1 200000 100000 0 -Sc .5 2 0 0 0 -Sc 1 4 0 0 0 -Sc 1.5 6 0
  0 0 -Sc 2 8 0 0 0 -Sc 2.5 10 0 0 0 -Sc 3 12 0 0 0 -Sc 3.5 14 0
  0 0 -Sc 4 16 0 0 0
```

```
./Seq-Gen.v1.3.3/source/seq-gen -mHKY -l 100000 -s .02 -p 483605
```

Supplemental Results

Bidirectional gene flow

We simulated bidirectional gene flow without selection between sister species in the first 2N generations after divergence and the most recent 2N generations at effective migration parameters of 0.001, 0.01, 0.1, 1, and 10. As for unidirectional gene flow, there was a faster than linear increase in the size of the divergent genome with all levels of early gene flow and with recent gene flow of up to 4Nm = 0.01 (Figure S1). Higher recent gene flow levels slowed (4Nm = 1) or eliminated (4Nm = 10) this increase.

Threshold value

To ensure that the observed shape of growth of the highly divergent portion of the genome was not an artifact of the choice of threshold value for designating windows as F_{ST} outliers, we compared 80th, 95th, and 99th percentile thresholds and the 95th percentile with all intervening windows above the 75th percentile as described in the Methods. All simulations have $N = 10^6$. Regardless of threshold level, recent gene flow (4Nm = 10) always produced a flat line, while the no gene flow scenario showed a faster than linear increase (Figure S2).

Number of fixed differences

We computed the numbers of fixed differences between sister species in each simulation to see how they change with divergence time and with gene flow/selection scenario. We expect this statistic to increase linearly with increasing time since divergence without gene flow or selection. This was the case for all early gene flow scenarios. The rate of increase in fixed differences slowed with higher recent gene flow without selection, and underwent a nonlinear increase followed by slower increase in high recent gene flow scenarios with selecion (Figure

S3).

F_{ST} versus divergence time

We compared both mean (over 500 bp windows) and global F_{ST} of simulated 100 kbp genomes versus divergence time between sister species (Figure S4). When no gene flow was present, F_{ST} increased at a diminishing rate, reaching a plateau at higher divergence times. High (4Nm = 10) recent gene flow produced no change in either mean or global F_{ST}. Predicted F_{ST} for the no migration scenario is shown in Figure S4. These values were obtained using the formula $F_{ST} = 1 - E(t_s)/E(t_d)$, where $E(t_s)$ is the expected time to coalescence for two alleles sampled from the same population and $E(t_d)$ is the expected coalescence time for two alleles sampled from different populations. From coalescent theory, $E(t_s) = 2N$ when there is no migration and N is the population size. $E(t_d)$ is the sum of the time backwards from the present to population splitting (4NT, where T is any positive number) and the expected time to coalescence before population splitting (2N, since we simulated populations that retained the same population size after splitting rather than being halved). Thus, predicted $F_{ST} = 1 - \frac{1}{2T+1}$.

Figures S5 and S6 show the change in distribution of window F_{ST} values with increasing divergence time in the no selection no gene flow and recent gene flow (4Nm = 10) scenarios respectively for a representative run of each simulation. They clearly show a rightward shift in F_{ST} over time when there is no migration, but show no change when high gene flow is simulated. Figures S7 and S8 likewise show the change in distribution of F_{ST} in simulations with divergent selection and no gene flow or high recent gene flow (4Nm = 10). The former resembles the same scenario without selection (Figure S5), while the latter shows a transition to higher F_{ST} values between 6N and 8N generations. Figures S9 zooms in on the extreme right tails of the distributions in Figures S5 to show the exponential increase in the number of outlier windows above the 95th percentile threshold.

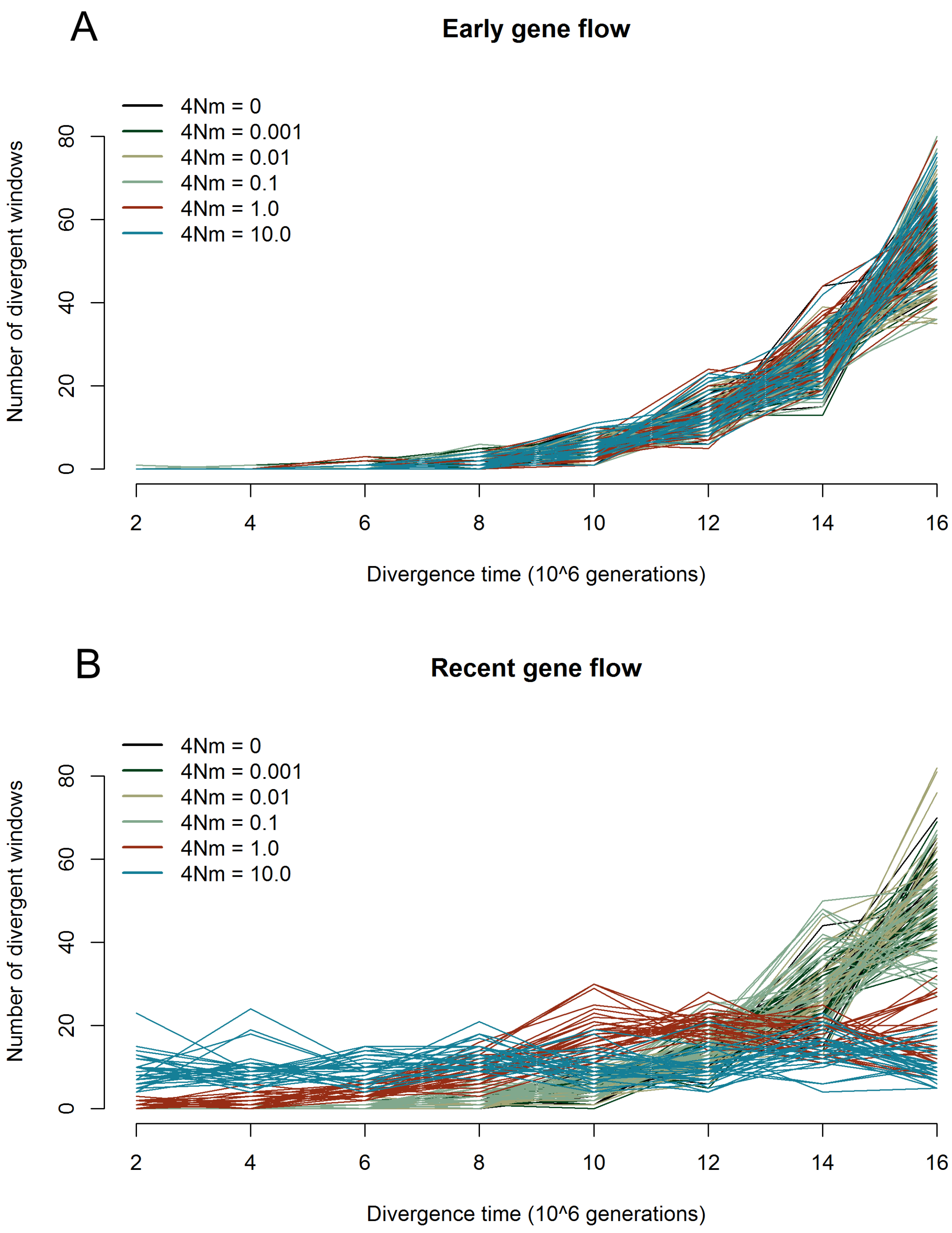

Figure S1: A: Divergent genome size (number of outlier windows) increases faster than linearly with divergence time when no gene flow or various levels of bidirectional early gene flow occurs. B: The highest levels of bidirectional recent gene flow homogenize the genome and prevent this increase. Each line represents a single simulation of eight between-species comparisons.

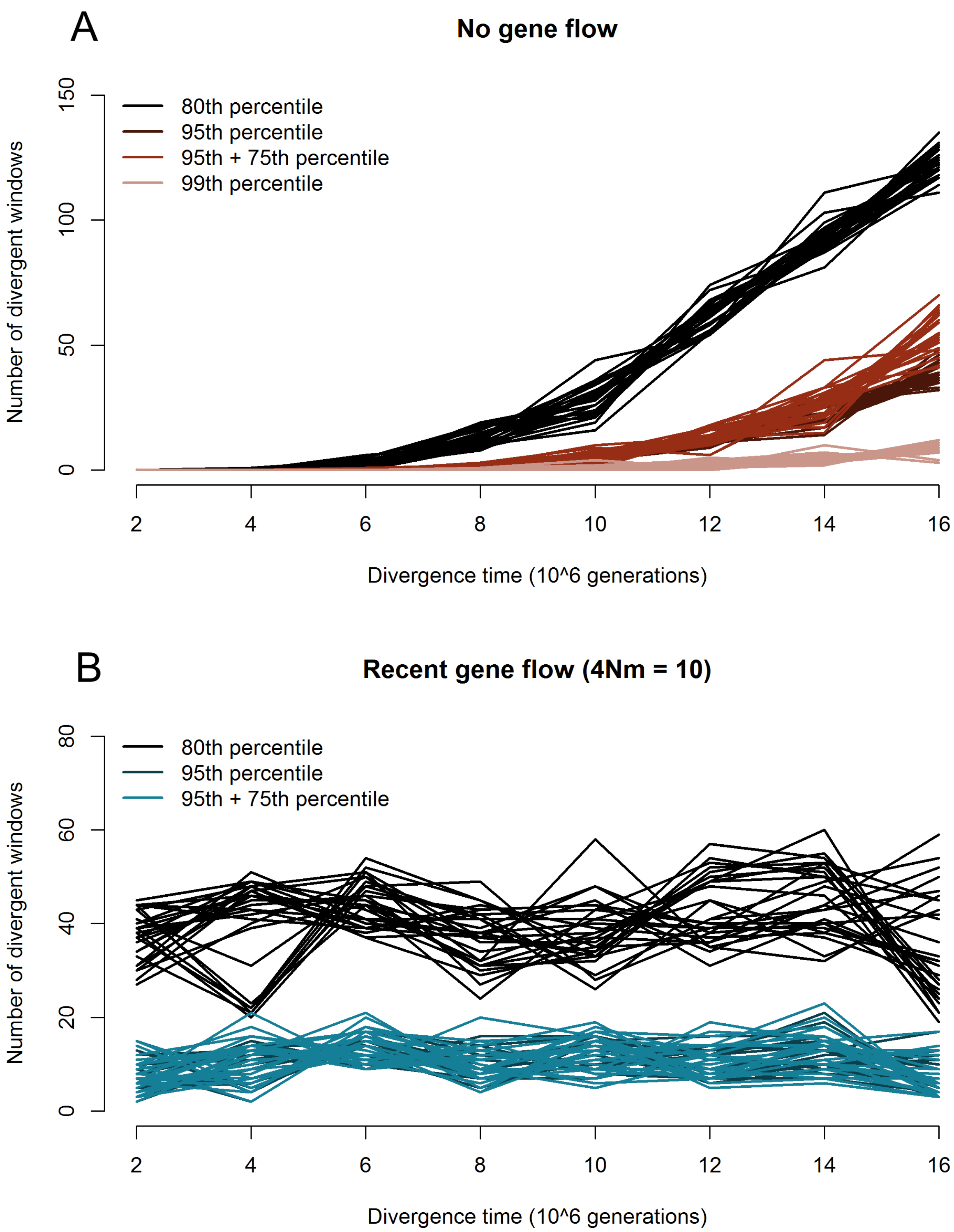

Figure S2: A: Changing the F_{ST} outlier threshold changes the steepness but not the shape (faster than linear) of the relationship between outlier size and divergence time in the no gene flow scenario. B: Changing the F_{ST} outlier threshold changes the number of windows classified as outliers but still shows a lack of growth in number of outliers with divergence time in the recent gene flow (unidirectional, 4Nm = 10) scenario.

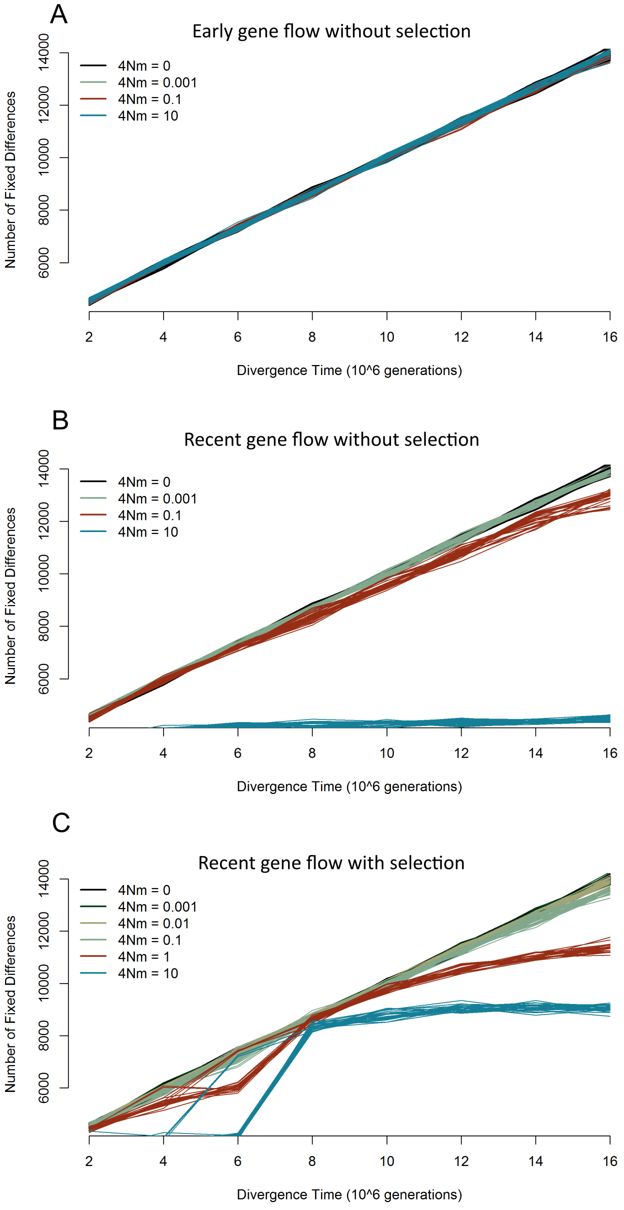

Figure S3: A,B: For simulations without selection, number of fixed differences between species pairs increases linearly with divergence time with early gene flow and, except at higher gene flow rates, with recent gene flow. C: With selection, the number of fixed differences increased linearly with low recent gene flow but nonlinearly with higher gene flow.

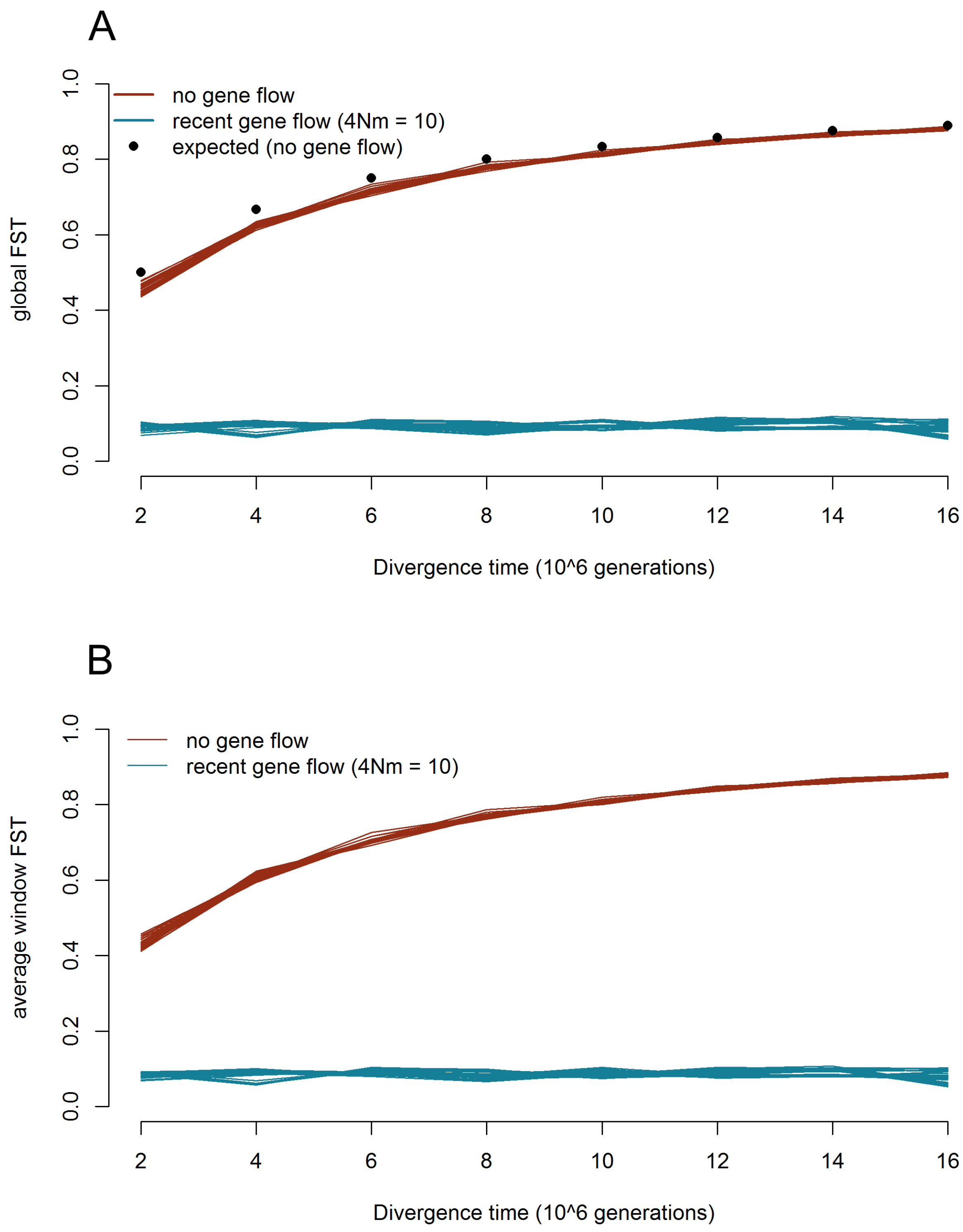

Figure S4: A: Genome-wide F_{ST} increases with divergence time when no gene flow is allowed, but stays constant if high recent gene flow (4Nm = 10) occurs. B: Mean window F_{ST} shows the same pattern.

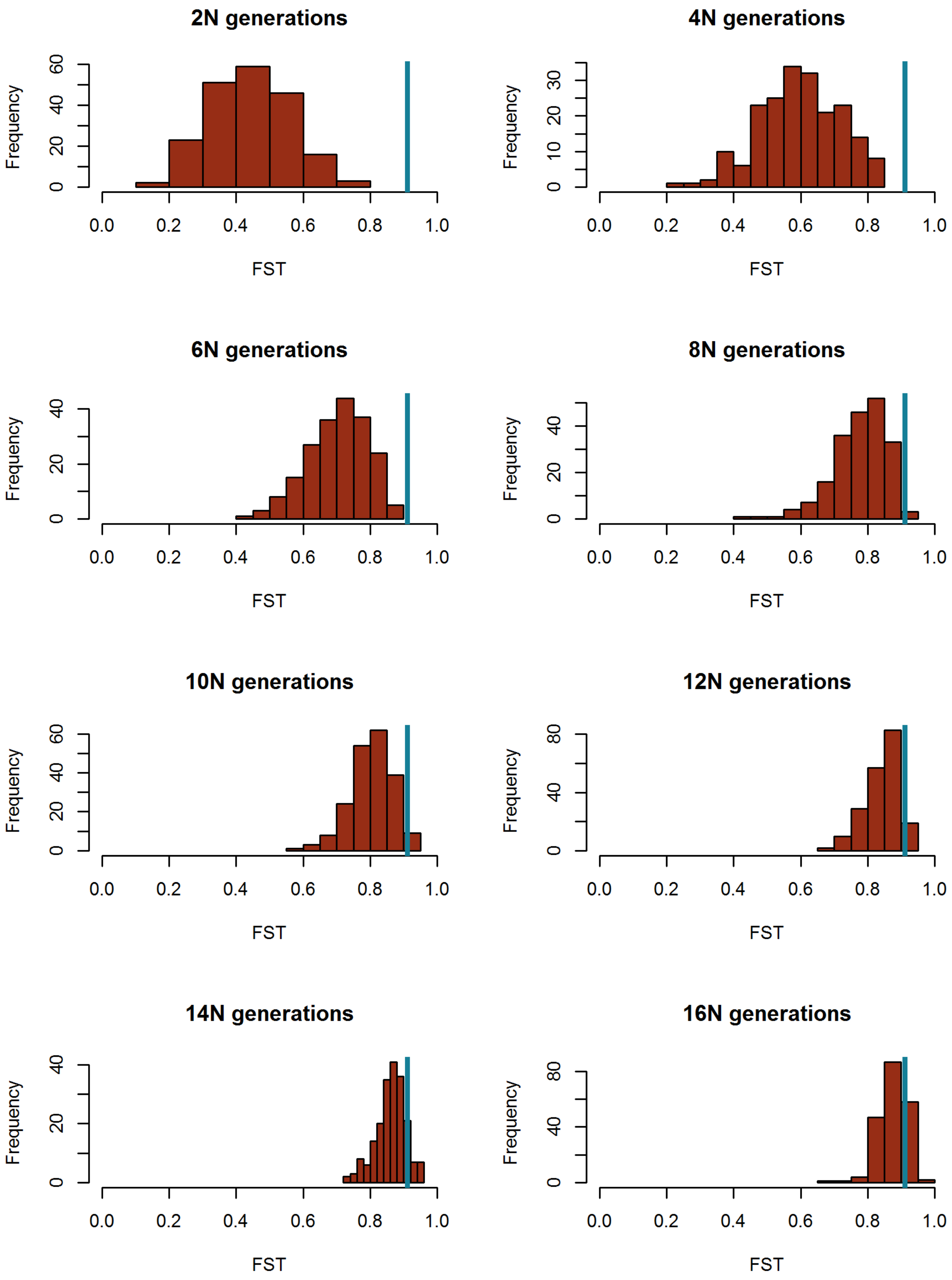

Figure S5: Distribution of F_{ST} values of 500 bp windows in a representative run of the no gene flow, no selection simulation for species pairs of different ages. Blue vertical lines indicate the 95% quantile of all F_{ST} values across all divergence times.

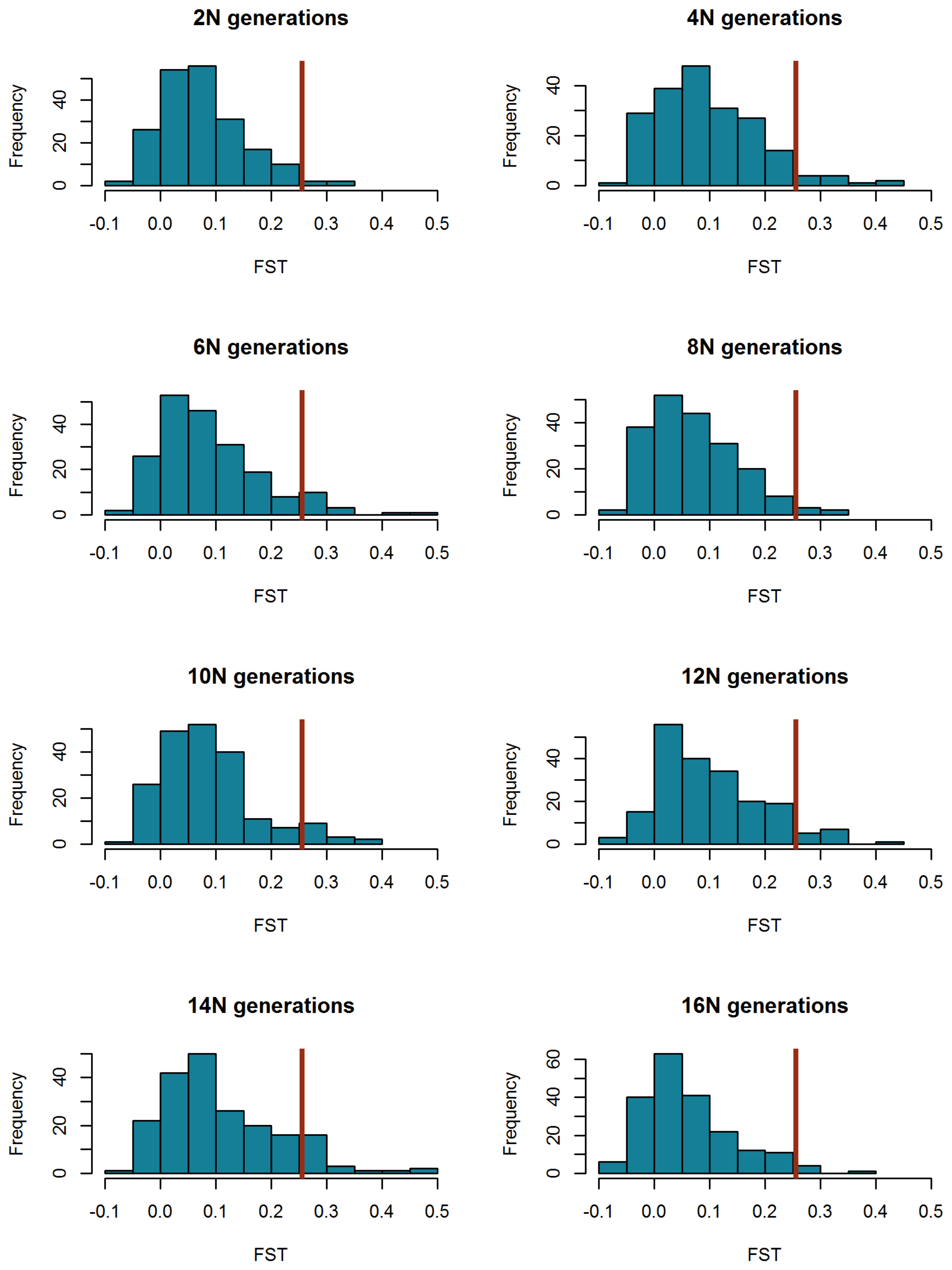

Figure S6: Distribution of F_{ST} values of 50[illegible] bp windows in a representative run of the simulations with the highest unidirectional gene flow ($4Nm = 10$) and no selection for species pairs of different ages. Red vertical lines indicate the 95% quantile of all F_{ST} values across all divergence times.

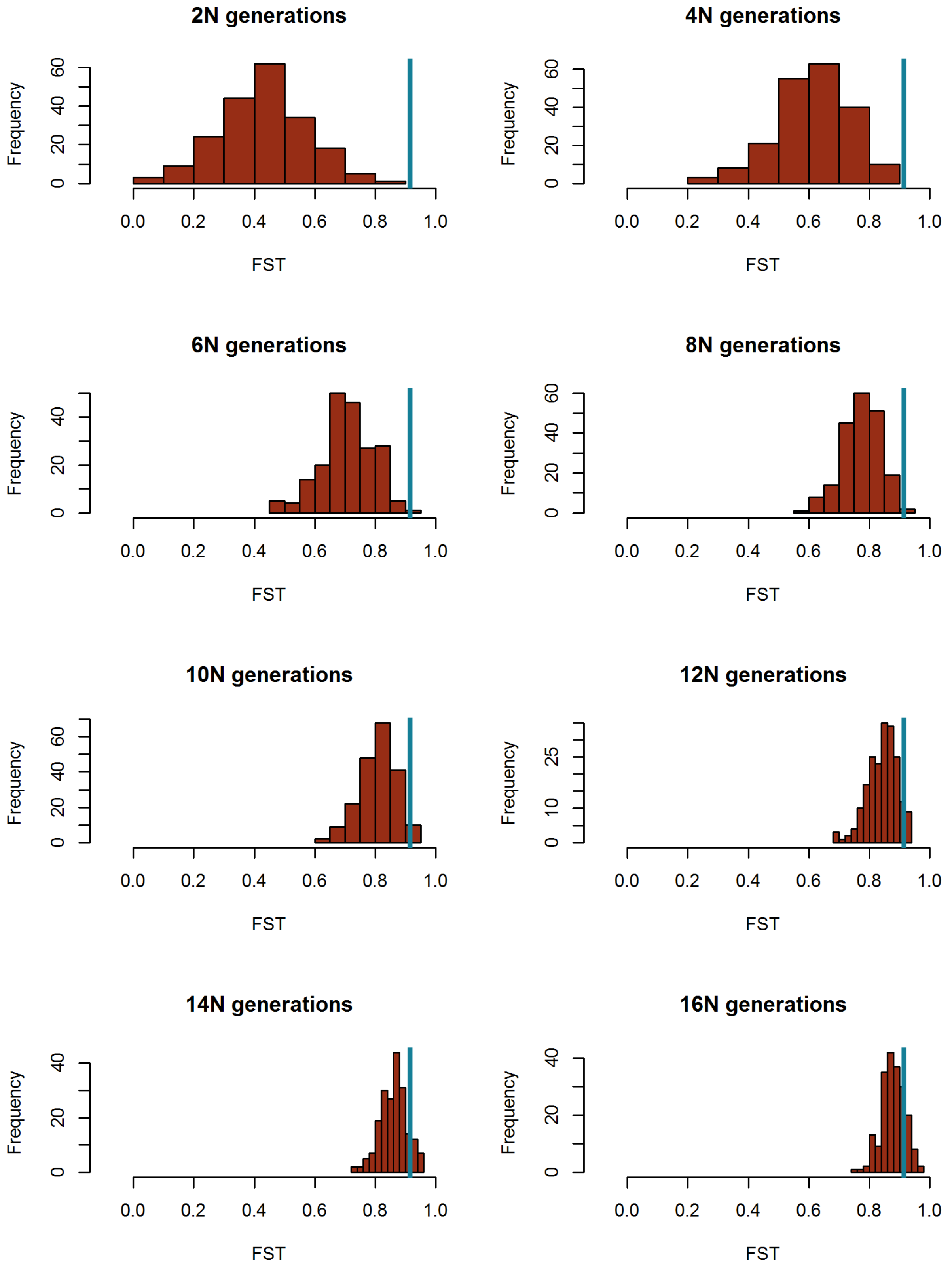

Figure S7: Distribution of F_{ST} values of 50$10^4$ bp windows in a representative run of the simulations with no gene flow and divergent selection for species pairs of different ages. Blue vertical lines indicate the 95% quantile of all F_{ST} values across all divergence times.

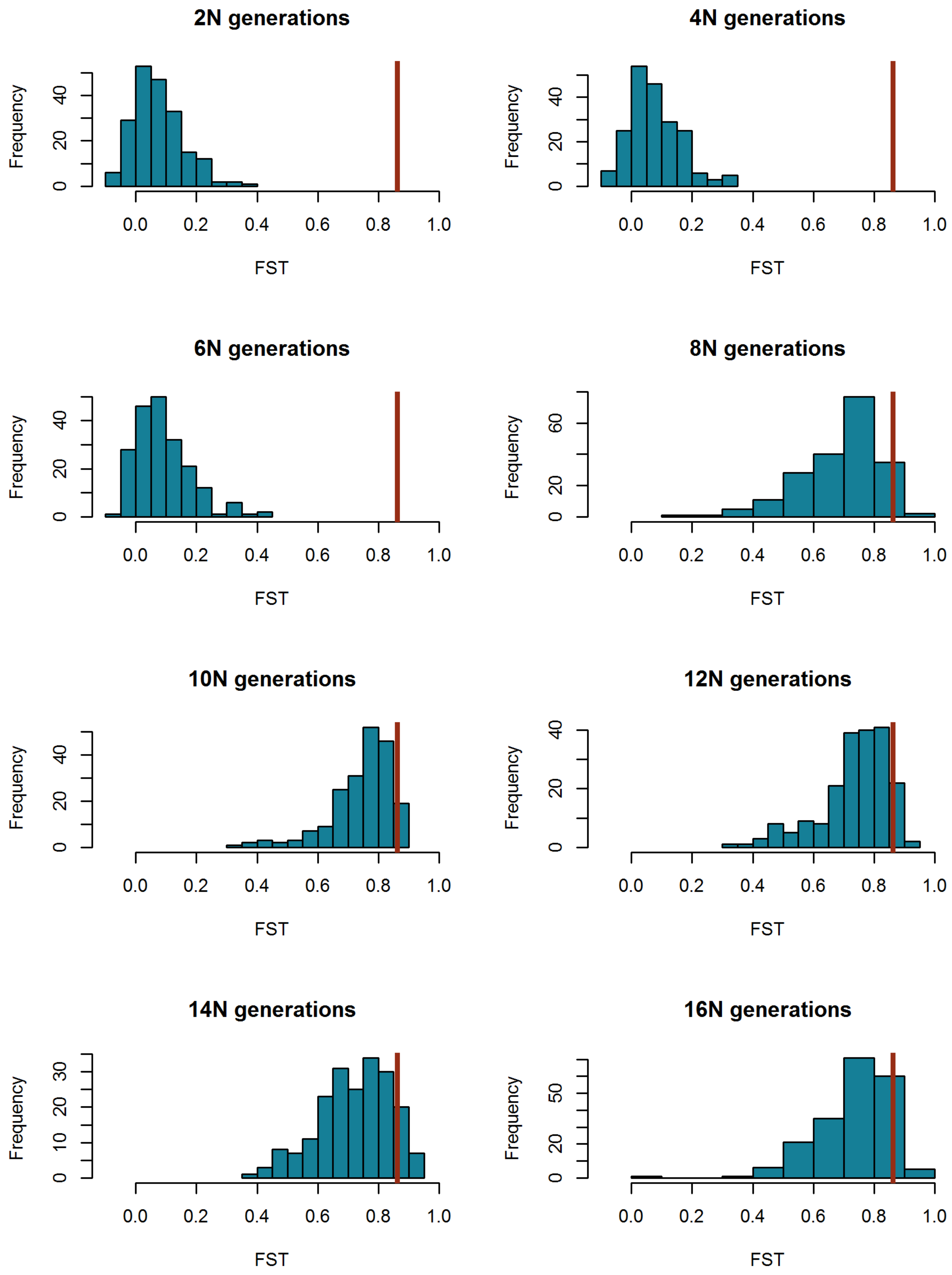

Figure S8: Distribution of F_{ST} values of 500 bp windows in a representative run of the simulations with the highest unidirectional gene flow (4Nm = 10) and divergent selection for species pairs of different ages. Red vertical lines indicate the 95% quantile of all F_{ST} values across all divergence times.

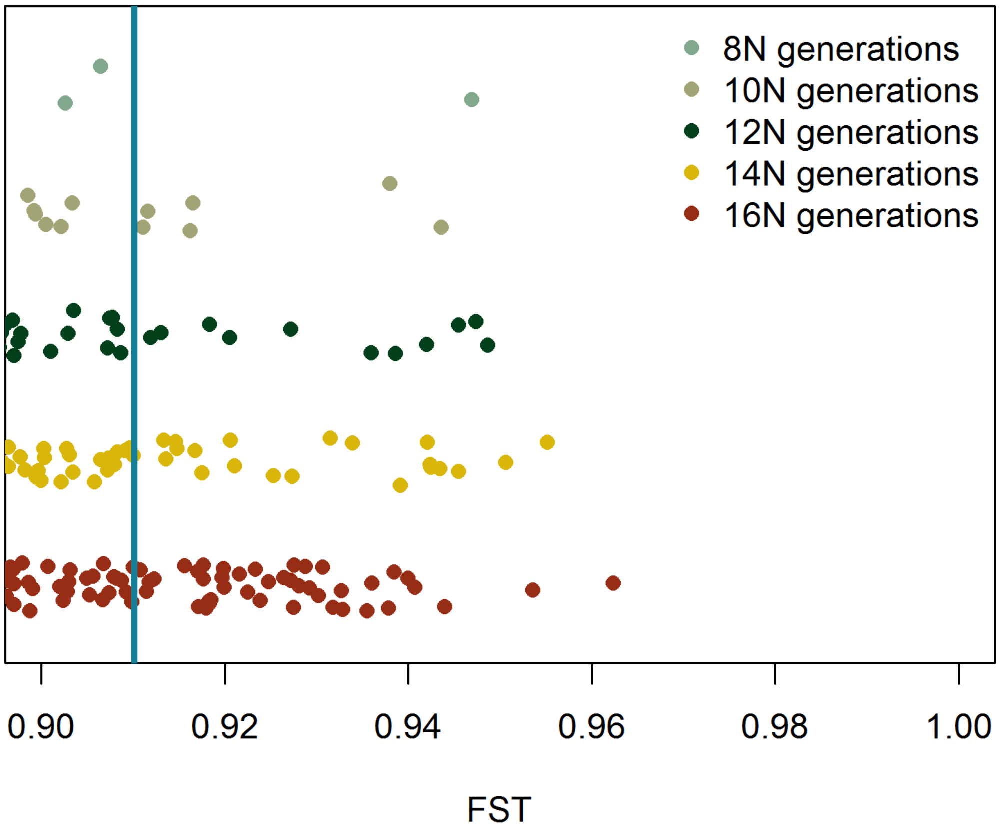

Figure S9: Upper tails of F_{ST} distributions showing number of windows above the 95th percentile threshold in each species pair. Each circle represents one 200bp window. Vertical blue line indicates the 95th percentile ($F_{ST} = 0.910$). Points to the right of this threshold were deemed outlier windows. Data are from a simulation with no gene flow or selection and $N = 10^6$. Three younger divergence times (2N, 4N, and 6N generations) are not shown because no windows exceeded the threshold.
